# Evaluation of ARK5 and SIRT3 expression in renal cell carcinoma and their clinical significance

**DOI:** 10.1186/s13000-023-01409-6

**Published:** 2023-11-23

**Authors:** Noha Elkady, Amira I. Aldesoky, Marwa Mohammed Dawoud

**Affiliations:** 1 Pathology Department, Faculty of Medicine, Menoufia University, Shibin El Kom, Menoufia 32511 Egypt; 2 Clinical Oncology and Nuclear Medicine Department, Faculty of Medicine, Menoufia University, Shibin El Kom, Menoufia Egypt

**Keywords:** ARK5, SIRT3, RCC, Prognosis, Response to therapy

## Abstract

**Background:**

Globally Renal Cell Carcinoma (RCC) represents 3% of malignant tumours in adults and 1.78% in Egypt. AMPK-related protein kinase 5 (ARK5) is mainly associated with a hypoxic microenvironment which is a feature of the major RCC subtypes. Additionally, it displays decreased mitochondrial respiration. SIRT3 is a mitochondrial deacetylase that modifies multiple mitochondrial proteins.

**Material and methods:**

Fifty eight cases of RCC, and 30 non-neoplastic cases (of End-Stage Kidney Disease (ESKD) were subjected to immunohistochemistry by ARK5 and SIRT3. The results of IHC were correlated together and correlated with the available clinicopathologic and survival data.

**Results:**

Although no significant difference was detected between RCC and ESKD groups regarding ARK5 expression, there was a significant association with RCC regarding H-score and nucleocytoplasmic expression (both *P* = 0.001). Also, SIRT3 was highly expressed in RCC in comparison to the ESKD group (H-score: *P* = 0.001). There were significant associations between nucleocytoplasmic ARK5 expression and higher tumour grade, low apoptotic and high mitotic indices, tumour extent, advanced tumour stage, and impaired response of tumours to chemotherapeutic drugs (*P* = 0.039, *P* = 0.001, *P* = 0.027, *P* = 0.011, *P* = 0.009, and *P* = 0.014 respectively). Moreover, the H score of ARK5 expression showed significant associations with tumour grade, apoptotic and mitotic indices, tumour extension, tumour stage, and response to therapy (*P* = 0.01, 0.035, 0.001, 0.004. 0.003 and 0.013). Regarding SIRT3 expression, it showed significant associations with apoptotic and mitotic indices, tumour extent, tumour stage and response to therapy (*P* = 0.022, 0.02, 0.042, 0.039 and 0.027). Interestingly, there was a highly significant correlation between the expression of ARK5 and SIRT3 (*P* = 0.009). Univariate survival analysis revealed a significant association between short survival duration and both nucleocytoplasmic expression of ARK5 and positive SIRT3 expression (*P* = 0.014 and 0.035).

**Conclusion:**

ARK5 and SIRT3 are overexpressed in RCC and associated with parameters of poor prognosis as well as short survival. Both seem to influence response to therapy in RCC. So, they could be new targets for therapy that may improve tumour response and patients’ survival. There is a postulated relationship that needs more extensive investigation.

## Introduction

Renal cell carcinoma represents 3% of malignant tumours in adults where it comprises 85% of all renal tumours [1]. In Egypt, renal cancer represents 11% of malignancies of the urinary system and 1.78% of all malignant tumours [2]. Histologically RCC is derived from cells lining the renal tubules. However, it comprises a heterogeneous disease with easily observed heterogeneous clinical outcomes. The fifth edition of the World Health Organization (WHO) classification of urogenital tumours (2022) included major revisions. It introduced new entities based on molecular classification [3]. Most cases of localized clear cell RCC (the most prevalent type of RCC) are cured by nephrectomy. Thus, chemotherapy has a limited role because its response is poor and about 30% of cases eventually develop metastases [4]. Thus, new biomarkers that can predict the response to chemotherapy and new target therapies are mandatory to potentiate the response to traditional modalities of treatment.

AMPK-related protein kinase 5 (ARK5) is a serine/threonine kinase that was recognized as one of the AMP-activated protein kinase (AMPK) family members [5]. ARK5 has reported a role in metastasis in various types of cancer such as colorectal (CRC) cancer, pancreatic cancer (PC), and squamous cell carcinoma [5–8]. Poor clinical prognosis prompted by ARK5 is mainly associated with a hypoxic microenvironment. This has been recognized in CRC where a close relationship with HIFs has been revealed [9]. To the best of our knowledge, no studies have investigated the expression of ARK5 in RCC despite the well-recognized activation of HIFs in a hypoxic environment, which is a feature of the major RCC subtypes [10]. Additionally, the α-subunits of the HIFs are the best-characterized targets of pVHL. Most sporadic ccRCC have somatic inactivation of VHL [11].

RCC displays increased aerobic glycolysis with decreased mitochondrial respiration due to constituent HIF-α expression [12]. It is synthesized as a 44 kDa peptide with an N-terminal sequence. SIRT3 is the primary NAD + -dependent mitochondrial deacetylase that modifies multiple mitochondrial proteins [13]. SIRT3 plays a crucial role in affecting or regulating various cellular processes, including metabolism, stress reactions, angiogenesis, cell proliferation, and apoptosis [13–15]. Lately, some studies have been conducted to investigate its role in tumorigenesis [14], including HCC [15], gastric cancer [16], and breast cancer (BC) [17]. Meanwhile, results are still controversial regarding SIRT3 prognostic role in RCC [18].

The aim of this study is to investigate the immunohistochemical expression of ARK5 and SIRT3 in a sample of RCC cases. This is to explore their proposed prognostic and predictive roles through correlation with clinicopathologic parameters, survival data, and resonse to therapy.

## Maternal and methods

This retrospective study has been conducted on 88 specimens of renal tissue including 58 cases of renal cell carcinoma (RCC), and 30 non-neoplastic cases (sections from end-stage kidney disease (ESKD). After obtaining the approval from Ethical Committee (11/2022PATH20) at the Faculty of Medicine Menoufia University, formalin-fixed, paraffin-embedded (FFPE) tissue blocks were obtained from the archive of the pathology department, Faculty of Medicine, Menoufia University the period from Jan 2017 and Dec 2021. Clinicopathologic data were retrieved from patients’ records, including gender, age, tumour size, response to chemotherapy and Overall Survival (OS).

From each representative paraffin block of each case, 4 μm-thick sections were cut, mounted on glass slides and stained by haematoxylin and eosin (H&E) stain in order to confirm the diagnosis and to evaluate pathological parameters of prognostic importance in RCC including histological type [19, 20]**,** tumour grade [19], pathologic tumour stage [21], and mitotic and apoptotic indexes.

### Tissue Microarray Technique (TMA)

The Tissue Microarray Constructing Technique (TMA) was accomplished for all studied cases after labelling carefully selected viable foci in H&E-stained sections of each case. The matching block of each case was labelled with a pen (Quick-Ray Tissue Microarray System) and bunched out at the selected foci. Three tissue cores (0.6 mm diameter) with a diameter of 1.5 microns from the donor block were punched using a manual tissue arrayer’s needle (Beecher Instruments, Silver Spring, MD, USA). Then the retrieved tissue cores were arrayed on a recipient block [22]. A map was created that shows the origin and location of each core. A core was taken from a normal foreign tissue and placed at specific positions throughout the block as a control. After constructing the TMA blocks, three 4μ thick sections were then cut from each block, 1 was mounted on glass slides for H&E staining and the other 2 sections were mounted on positively charged slides to be used for immunohistochemical staining*.*

### Immunohistochemical staining

The steps of immunohistochemistry followed the protocol conducted using the fully automated immunohistochemical machine (DAKO). The method used for immunostaining was the streptavidin–biotin amplified system. Antigen retrieval was performed using citrate buffer PH 6.0. The used primary antibodies included ARK5 Ab (Rabbit polyclonal antibody GTX53533, 400um,1:100, GeneTex, USA), and SIRT3 Ab (Rabbit polyclonal antibody A17113, 0.1 ml con, 1:50, ABclonal, Woburn, USA). Positive control for ARK5 was breast carcinoma, and for SIRT3 was colorectal carcinoma. Negative controls were prepared by the omission of the step of primary antibody.

### Interpretation of immunostaining

Sections immunostained by ARK5 and SIRT3 in malignant and non-neoplastic groups were evaluated and scored semi-quantitatively by two pathologists (N.K and M.D.) independently and blinded to the clinical parameters. Cases were considered positive when any number of cells showed brown staining. Subcellular localization of the expression was also evaluated. The expression was semiquantitatively scored using the H score where the intensity of staining was evaluated (in reference to the positive and negative control slides) was considered as 0 = negative (no staining), 1 = mild (faint light brown staining), 2 = moderate (pale brown staining) and 3 = strong (dark brown staining) then the percentage of cells with positive expression was also assessed by dividing the number of positive cells by the number of the whole cells into 10 random fields. H score was calculated by multiplying the intensity by the percentage of positive cells (H = 0–300 [23]). Each one of the examining pathologists has subjectively determined the intensity of staining and percent of positive cells then the consensus results of both were taken.

### Statistical analysis

All statistical analyses were performed using Statistical Product and Service Solutions (SPSS) software version 22 (SPSS Inc., Chicago, IL,USA): Statistical tests included: Descriptive statistics using percentage, and mean and standard deviation (SD) and Analytic statistics tests including Student's t-test (t), Mann–Whitney test (U), Kruskal–Wallis test (KW), Chi-square test (X^2^) and fisher's exact test (FE) [24].

Overall survival (OS) was estimated from the date of diagnosis until the date of death or the date of the last contact. Univariate survival data analysis was done using the Kaplan–Meier curve and the log-rank test to compare between groups followed by multivariate survival analysis using Cox regression testing to elicit the independent survival predictor [25].

Differences were considered: Highly significant (HS) when (*P* < 0.01), statistically significant (S) when (*P* ≤ 0.05), and not significant (NS) when (*P*> 0.05) [24].

## Results

Males slightly predominate in the malignant cases where they represent 62.1%. Patients’ ages ranged from 33 to 75 years old with a mean age of 57.3. Clear cell type was the most prominent histological type representing 55.2% of cases and nearly half of the cases were grade 2. About seventy percent of cases were early stage where tumours were limited to the kidney and only 3.4% were extended beyond Gerota’s fascia. Lymph node metastasis was found in only 2 cases. More than half of the cases (56.8%) showed partial response to chemotherapy and only 13.6% showed a complete response (Table 1).

**Table 1:** Clinicopathological data of the studied cases

	Total number
Gender	
Male	36 (62.1%)
Female	22 (37.9%)
Age	
Mean ± SD	57.3 ± 9.4
Histological type clear	32 (55.2%)
Papillary	7 (12.1%)
Chromophobe	16 (27.6%)
Others	3 (5.2%)
Grade	
1	15 (25.9%)
2	21 (36.2%)
3	14 (24.1%)
4	8 (13.8%)
Apoptosis	
Mean ± SD	2.5 ± 0.88
Mitosis	
Mean ± SD	2.8 ± 1.1
Tumour extent	
T1	20 (34.5%)
T2	21 (36.2%)
T3	15 (25.9%)
T4	2 (3.4%)
LN	
Negative	56 (96.6%)
Positive	2 (3.4%)
Stage	
I	20 (34.5%)
II	20 (34.5%)
III	16 (27.6%)
IV	2 (3.4%)
Response to chemotherapy	
No	13 (29.5%)
Partial	25 (56.8%)
Complete	6 (13.6%)

### Immunohistochemical expression of ARK5 and SIRT3 

**Fig. 1:**
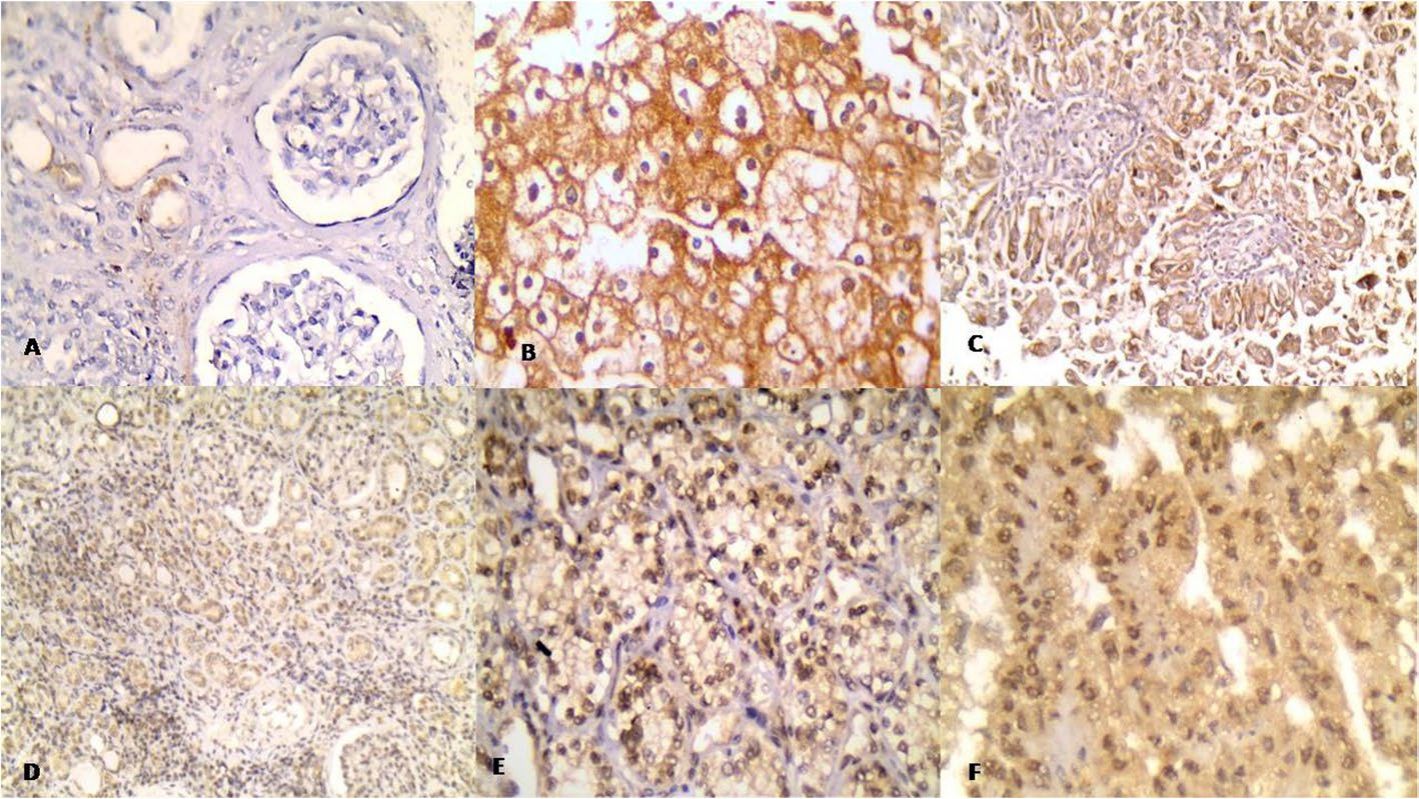
Immunohistochemical expression of ARK5 and SIRT3 **A** Mild focal cytoplasmic SIRT3 expression in non-neoplastic renal tissue. **B** Diffuse strong cytoplasmic expression of SIRT3 in clear cell renal cell carcinoma. **C** Diffuse strong cytoplasmic expression of SIRT3 in papillary renal cell carcinoma. **D** Mild cytoplasmic ARK5 expression in non-neoplastic renal tissue. **E** Diffuse strong nucleo-cytoplasmic expression of ARK5 in clear renal cell carcinoma. **F** Diffuse strong nucleo-cytoplasmic expression of ARK5 in papillary renal cell carcinoma

Fifty-five cases of RCC showed ARK5 expression where 19 cases showed cytoplasmic expression and 36 showed nucleocytoplasmic expression meanwhile 24 non-neoplastic cases showed cytoplasmic ARK5 expression (Table 2) (Fig. 1).

**Table 2:** Expression of ARK5 and SIRT3 in RCC and non-neoplastic cases

	**RCC** **(** ***n*** ** = 58)** **(%)**	**Non-neoplastic** **(** ***n*** ** = 30)** **(%)**	**Test**	***P*** ** value**
**Expression**			Fisher Exact Test	0.057
ARK5 negative	3 (5.2%)	6 (2%)		
ARK5 positive	55(94.8%)	24 (98%)		
**Intracellular localization**			Fisher Exact Test	0.001**
ARK5 cytoplasmic	19 (34.5%)	24 (100%)		
ARK5 nucleocytoplasmic	36 (65.4%)	0 (00%)		
**H-score**	164.5 ± 55.651	113.3 ± 65.7	Mann–Whitney	0.001**
**Expression**			Fisher Exact Test	0.002*
SIRT3 negative	14 (24.14%)	18 (6%)		
SIRT3 positive	44 (75.9%)	12 (94%)		
**H- score**	134.6 ± 81.2	59.7 ± 76.3	Mann–Whitney	0.001**

^*^significant, **highly significant

SIRT3 expression was detected in 44 cases of RCC and 12 non-neoplastic cases. SIRT3 positivity appeared as cytoplasmic brown staining (Table 2) (Fig. 1).

The nucleocytoplasmic expression of ARK5 and the cytoplasmic SIRT3 expression showed significant associations with RCC cases when compared to non-neoplastic cases (*P* = 0.001 and 0.002).

A statistically significant difference was also observed between RCC cases and non-neoplastic cases as regards the H score of both ARK5 and SIRT3 expression (*P* = 0.001 and 0.001) where a higher H score was significantly associated with malignant cases (Table 2).

### Correlation between ARK5 expression and clinicopathological parameters of RCC cases

The present study revealed a significant association between nucleocytoplasmic ARK5 expression and higher tumour grade (0.039). In addition, low apoptotic and high mitotic indices were significantly associated with nucleocytoplasmic ARK5 expression (*P* = 0.001 and 0.027). Moreover, significant associations were found between nucleocytoplasmic ARK5 expression and tumour extent and advanced tumour stage (*P* = 0.011 and 0.009) where larger tumour size and those extending to perinephric fat, renal vessels and beyond Gerrota’s fascia showed nucleocytoplasmic ARK5 expression. Moreover, nucleocytoplasmic ARK5 expression was significantly associated with the impaired response of tumours to chemotherapeutic drugs (*P* = 0.014) (Table 3).

**Table 3:** Correlation of ARK5 intracellular localization in RCC with clinicopathological parameters

	Cytoplasmic (*n* = 19) (%)	Nucleocytoplasmic (*n* = 36) (%)	Test	*P* value
Gender			Fisher Exact Test	0.39
Male	10 (52.63%)	24 (66.7%)		
Female	9 (47.36%)	12 (33.3%)		
Histological type clear	10 (52.6%)	19 (52.8%)	Chi square	0.67
Papillary	2 (10.5%)	5 (13.9%)		
Chromophobe	5 (26.3%)	11 (30.6%)		
Others	2 (10.5%)	1 (2.8%)		
Grade				
1	8 (42.1%)	4 (11.1%)	Chi-square	0.039*
2	7 (36.8%)	14 (38.9%)		
3	2 (10.5%)	12 (33.3%)		
4	2 (10.5%)	6 (16.7%)		
Apoptosis	3.05 ± 0.91	2.19 ± 0.71	Mann–Whitney	0.001**
Mitosis	2.4 ± 0.9	3.1 ± 1.2	Mann–Whitney	0.027*
Tumour extent				
T1	11 (57.9%)	6 (16.7%)	Chi-square	0.011*
T2	6 (31.6%)	15 (41.7%)		
T3	2 (10.5%)	13 (36.1%)		
T4	0 (00%)	2 (5.6%)		
LN				
Negative	19 (100%)	34 (94.4%)	Fisher Exact Test	0.54
Positive	0 (00%)	2 (5.6%)		
Stage				
I	11 (57.9%)	6 (16.7%)	Chi square	0.009*
II	6 (31.6%)	14 (38.9%)		
III	2 (10.5%)	14 (38.9%)		
IV	0 (00%)	2 (5.6%)		
Response to chemotherapy				
No	2 (10.5%)	11 (30.6%)	Chi-square	0.014*
Partial	8 (42.1%)	17 (47.2%)		
complete	5 (26.3%)	1 (2.8%)		

*Significant, **Highly significant

Moreover, the H score of ARK5 expression showed significant associations with tumour grade, apoptotic and mitotic index, tumour extension, stage and response to therapy (*P* = 0.01, 0.035, 0.001, 0.004. 0.003 and 0.013) where the higher the H score, the higher the tumour grade, the lower the apoptotic index, the higher the mitotic index, the larger the tumour extent, the higher the stage and the lesser the response to chemotherapy (Table 5).

### Correlation between SIRT3 expression and clinicopathological parameters of RCC cases

SIRT3 expression showed significant associations with apoptotic and mitotic index, tumour extent, tumour stage and response to therapy (*P* = 0.022, 0.02, 0.042, 0.039 and 0.027) as positive SIRT3 expression was associated with the lower apoptotic index, higher mitotic index, more tumour extension into renal parenchyma and perirenal fascia, advanced tumour stage and worse response to chemotherapy (Table 4).

**Table 4:** Correlation of SIRT3 expression in RCC with clinicopathological parameters

	Negative (*n* = 14)(%)	Positive (*n* = 44)(%)	Test	*P* value
Gender			Fisher Exact Test	0.53
Male	10 (71.4%)	26 (59.1%)		
Female	4 (28.6%)	18 (40.9%)		
Histological type			Chi square	0.21
Clear	9 (64.3%)	23 (52.2%)		
Papillary	3 (21.4%)	4 (9.1%)		
Chromophobe	1 (7.1%)	15 (34.1%)		
Others	1 (7.1%)	2 (4.5%)		
Grade				
1	4 (28.6%)	11 (25%)	Chi-square	0.17
2	8 (57.1%)	13 (29.5%)		
3	1 (7.1%)	13 (29.5%)		
4	1 (7.1%)	7 (15.9%)		
Apoptosis	2.93 ± 0.83	2.32 ± 0.86	Mann–Whitney	0.022*
Mitosis	2.1 ± 1.1	2.97 ± 1.1	Mann–Whitney	0.02*
Tumour extent				
T1	9 (64.3%)	11 (25%)	Chi-square	0.042*
T2	4 (28.6%)	17 (38.6%)		
T3	1 (7.1%)	14 (31.8%)		
T4	0 (00%)	2 (4.5%)		
LN				
negative	14 (100%)	42 (95.4%)		0.57
Positive	0 (00%)	2 (4.5%)		
Stage			Chi-square	0.039*
I	9(64.3%)	11(25%)		
II	4 (28.6%)	16 (36.4%)		
III	1 (7.1%)	15 (34.1%)		
IV	0 (00%)	2 (4.5%)		
Response to chemotherapy				
No	1 (7.1%)	12 (27.2%)	Chi square	0.027*
Partial	7 (50%)	18 (40.9%)		
Complete	4 (28.6%)	2 (4.5%)		

*Significant

Furthermore, significant associations were found between H score of SIRT3 expression and tumour grade, apoptotic and mitotic index, tumour extent, tumour stage and tumour response to therapy (*P* = 0.037, 0.016, 0.002, 0.001, 0.001 and 0.037) where higher tumour grade, low apoptosis, higher cellular proliferation, larger tumour size, extension to renal vessels and perirenal fascia, advanced tumour stage and worse response to chemotherapy were associated with higher H score. (Table 5).

**Table 5:** Correlation between H score of ARK5 and SIRT3 expression and clinicopathological parameters

	ARK5- H -score Mean ± SD	SIRT3- H -score Mean ± SD	Test	*P* value
Gender				
Male	163.7 ± 57.7	128.6 ± 83.2	Mann–Whitney	P1 = 0.85 P2 = 0.82
Female	165.7 ± 53.2	144.5 ± 78.9		
Histological type clear	154.8 ± 66.3	128.4 ± 88.4	Kruskal–Wallis	P1 = 0.11 P2 = 0.13
Papillary	160.0 ± 30.0	77.1 ± 77.8		
Chromophobe	191.6 ± 31.8	172.5 ± 39.2		
Others	133.3 ± 28.8	133.3 ± 115.9		
Tumour grade				
1	118.0 ± 69.9	114.67 ± 74.7	Kruskal–Wallis	P1 = 0.01* P2 = 0.037*
2	175.2 ± 38.68	115.7 ± 88.8		
3	187.86 ± 31.67	162.86 ± 64.97		
4	182.5 ± 53.18	172.5 ± 83.96		
Apoptosis	164.48 ± 55.6	134.66 ± 81.2	Pearson	**P1 = 0.035** P2 = 0.016*
Mitosis	164.5 ± 55.6	134.66 ± 81.2	Pearson	P1 = 0.001** P2 = 0.002*
Tumour Extent				
1	130.5 ± 67.2	92.5 ± 79.5	Kruskal–Wallis	P1 = 0.004* P2 = 0.001**
2	177.1 ± 37.0	133.8 ± 72.1		
3	182.0 ± 37.8	187.0 ± 67.8		
4	240.0 ± 21.2	230.0 ± 42.4		
Stage				
I	130.5 ± 67.2	92.5 ± 79.5	Kruskal–Wallis	P1 = 0.003* P2 = 0.001**
II	174.0 ± 35.0	132.5 ± 73.7		
III	185.6 ± 39.3	176.9 ± 65.7		
IV	240.0 ± 21.2	240.0 ± 42.4		
Response to chemotherapy				
No	200.4 ± 23.5	183.1 ± 62.1	Kruskal–Wallis	P1 = 0.013* P2 = 0.037*
Partial	177.4 ± 40.9	123.2 ± 82.5		
Complete	141.7 ± 43.1	53.3 ± 88.5		
ARK5 and SIRT3	164.5 ± 55.651	134.6 ± 81.2	Pearson correlation (R) 0.342	0.009*

P1 = ARK5 H-score, P2 = SIRT3 H-score, * significant, ** highly significant

Interestingly, there was a significant correlation between the expression of ARK5 and SIRT3 (*P* = 0.009) in the studied cases. (Table 5).

### Survival analysis

**Fig. 2:**
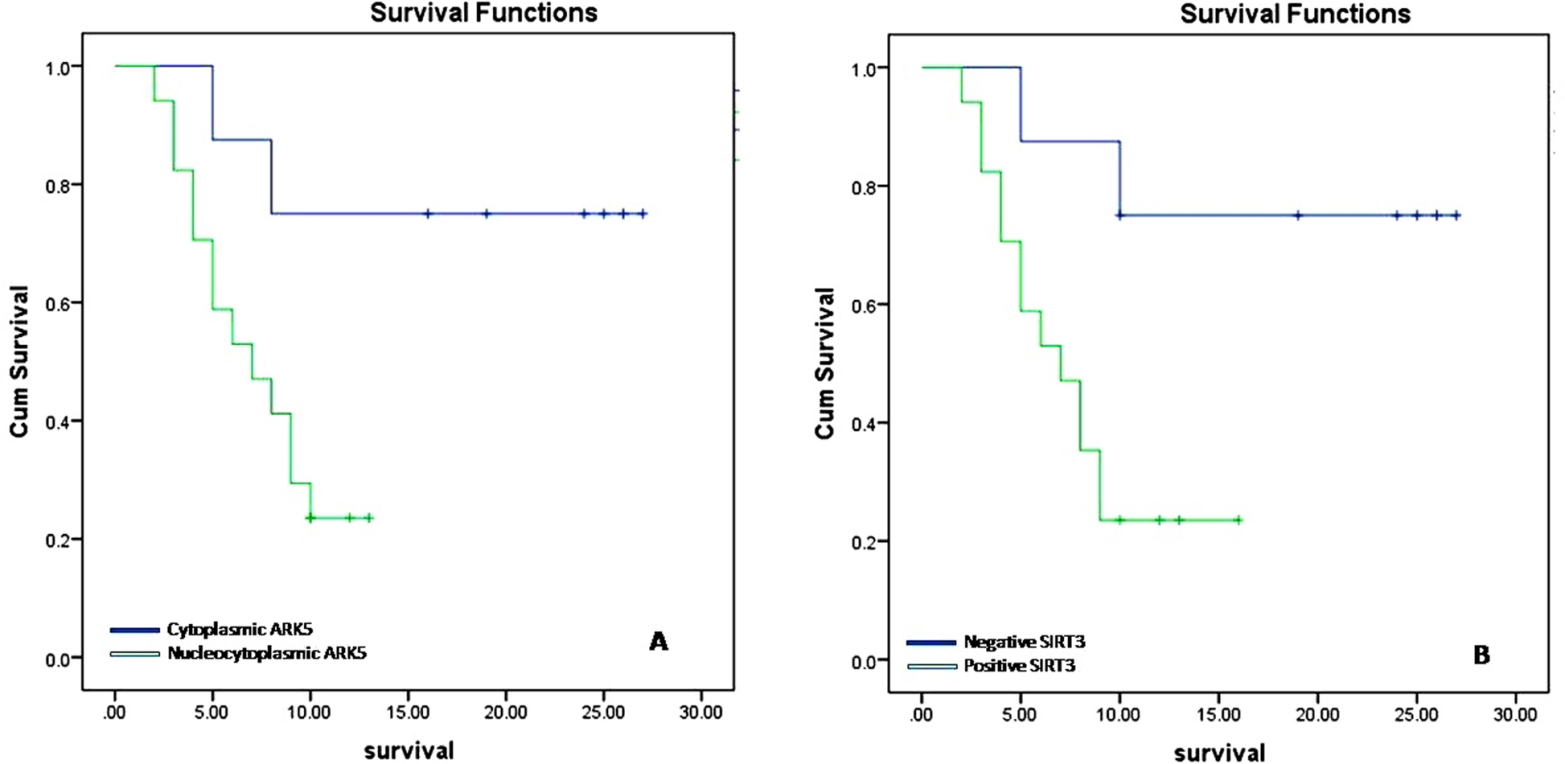
Kaplan Meier survival curves for clear RCC patients showing decreased survival duration for cases with nucleocytoplasmic ARK5 expression (**A**) and SIRT3 positivity (**B**)

Kaplan Meier univariate survival analysis of all renal cell carcinoma cases was done using the Log Rank test, it revealed significant associations between short survival duration and both nucleocytoplasmic expression of ARK5 and positive SIRT3 expression (*P* = 0.014 and 0.035). Detailed Kaplan Meier univariate survival analysis of each type of RCC revealed that nucleocytoplasmic ARK5 expression and SIRT3 positivity have a significant negative impact on the survival of patients with clear RCC (*P* = 0.024 and 0.016) (Fig. 2) while they do not have any effect on the survival of patients with papillary RCC (*P* = 0.99 and 0.94) nor chromophobe RCC (*P* = 0.67 and 0.59).

Multivariate survival analysis using the Cox regression test revealed that response to chemotherapy is the only independent variable that affects the survival of patients with renal cell carcinoma (p < 0.001).

## Discussion

RCC is now the 7th leading cancer type in men in the US and the incidence has been steadily rising by 2–4% each year with a 2:1 male-to-female ratio. Cigarette smoking, obesity, hypertension and/or related medications have been implicated as risk factors in addition to individuals at an advanced stage of chronic kidney disease (CKD) on long-term dialysis. Approximately 2–4% of RCC is hereditary and some of the predisposing genes have been identified and are available for genetic screening [26].

The most common histological type is clear cell carcinoma, It is also called conventional RCC, it represents 75–80% of RCC. Papillary (10–15%), chromophobe (5%) and other rarer forms including collecting duct carcinoma (< 1%) comprise the remainder. An individual tumour can have a mixture of multiple histological types [27].

Malignant tumors are formed of highly proliferating cells which need energy for their survival meanwhile, malignant tumors try to make their own vasculature but still, the angiogenesis is inadequate resulting in relative hypoxia and glucose deficiency. So, tumour cells try to adapt to this harsh environment by modifying energy metabolism, cell cycle and expression of hypoxia-associated molecules.

AMPK-related protein kinase 5 (ARK5) is one of the serine/threonine kinases, it is one of the metabolite-sensing protein kinase family. ARK5 is one of the essential molecules for oncogenesis, tumour cell proliferation, survival, invasion and metastasis. During stress, ATP is depleted leading to activation of ARK5 which regulates different molecules such as glucose transporters, acyl-CoA carboxylase and HMG-CoA reductase leading to shifting the metabolism from anabolism to catabolism with further protection of tumour cells [28, 29].

This study revealed that ARK5 is overexpressed in renal cell carcinoma where it showed nucleocytoplasmic localization. Other studies have also found that ARK5 expression was overexpressed in many cancers such as oesophagal carcinoma, ovarian carcinoma and multiple myeloma [30–32].

In addition, ARK5 nucleocytoplasmic expression and higher H score showed significant associations with high-grade RCC, wider tumour extension and advanced tumour stage.

This is in concordance with a study done on colorectal carcinoma which revealed that ARK5 expression was associated with tumour aggressiveness, progression, invasion and metastasis [7]. The same results were also documented by Kusakai et al., 2004 who reported the association between ARK5 expression and invasion and metastasis of colorectal [7].

High-grade tumors with high cellular proliferation rapidly grow in size which exceeds their blood supply exposing the tumour to hypoxic conditions and a relative decrease in glucose concentration this in its turn stimulates a hypoxia-related response. HIF-1 is one of the most important molecules that is overexpressed in solid tumours during hypoxia and it helps them to adapt to these unfavorable conditions. HIF-1 in its turn activates a group of hypoxia-related genes such as VEG, ARK5 and glycolysis-associated genes that help tumour cells to adapt and survive [7].

ARK5 promotes tumour cell motility increasing their ability for invasion and metastasis. This can be mediated through different mechanisms, one of them being the stimulation of the Akt pathway. Another mechanism is the induction of IGF-1 with further disruption of adherence junction and localization of actin to the moving part of the cell. Tumour cell invasion of the surrounding stroma with further metastasis can also be mediated by promoting MMP-2, MM-9 and MT1-MMP translation. ARK5 also induces EMT through the regulation of mTOR/p70S6k pathway, Slug and SIP-1 signalling [33–35].

ARK5 expression in tumour cells showed a significant association with a low apoptotic index and a higher mitotic index. The same results were described by Kusaki et al., 2004 in colorectal carcinoma where they found that the apoptotic index is low and the proliferation rate is high in cases expressing ARK5 [7].

ARK5 expression prevents cellular apoptosis via different pathways. It suppresses death receptor-induced cell death. Moreover, ARK5 decreases apoptosis via inhibition of Fas/FasL, caspase 6 and caspase 8 [36, 37].

Nucleocytoplasmic expression of ARK5 in the studied cases was also associated with decreased tumour response to chemotherapy and the higher the H score of its expression, the worse the response.

The same result was observed by Xu et al., 2016 who found that ARK expression was associated with Gemcitabine and Doxorubicin resistance in pancreatic and hepatocellular cancer [38].

ARK5 can mediate drug resistance through its antiapoptotic role. In addition, it acts as an inducer of tumour epithelial-mesenchymal transition (EMT) via activation of Twist gene and Hedgehog signaling pathway which is a main pathway for EMT. ARK5 can also activate the active pumping out of the chemotherapeutic drug which decreases its concentration inside the cell making it less effective [39, 40].

Sirtuins (SIRT) are the mammalian orthologs of the *Saccharomyces cerevisiae* silent information regulator proteins. They act as NAD-dependent deacetylases or ADP-ribosyl transferases. They include 7 members with different subcellular localization and different cellular targets. SIRT3 is located mainly in the mitochondria but it can translocate to the nucleus with further modulation of other targets [41, 42].

Regarding SIRT3 expression in the studied cases, it showed significant associations with lower apoptotic and higher mitotic index, larger tumour extent and advanced tumour stage. These results have been observed by other researchers who noticed that overexpression of SIRT3 is associated with large tumour size, lymph node metastasis and shorter overall survival of prostatic and breast cancer [43–45].

Evasion of apoptosis can be mediated via SIRT3 modulation of different signaling pathways. SIRT3 promotes tumour cell survival via the activation of the telomerase enzyme which allows unlimited tumour cell replication with further tumour growth [41].

SIRT3 maintains mitochondrial stability and promotes an optimal level of reactive oxygen species (ROS) essential for the survival of tumour cells which is achieved via the regulation of metabolism and cellular glycolysis [46]. Moreover, SIRT3 was found to induce anoikic resistance through regulation of the death/survival Fas/RIP/FAK pathway [47]. In addition, SIRT3 can maintain tumour cell survival via interaction with permeability transition pore (cyclophilin D) [48].

SIRT3 expression in the studied cases was significantly associated with impaired tumour response to chemotherapy. The same results have also been observed as a significant association between SIRT3 expression and chemoresistance in colorectal cancer was detected [49] and acute myeloid leukaemia [50].

SIRT3 can induce chemoresistance via diverse mechanisms. Besides its role as an antiapoptotic, SIRT3 overexpression enhances the balance of ROS production in tumour cells which renders them resistant to chemotherapeutic drugs. Moreover, SIRT3 can also potentiate drug resistance via dysregulation of mitochondrial oxidative phosphorylation, deacetylation of SOD2 and modulation ofPGC-1a [51, 52].

In addition, SIRT3 promotes tumour cell autophagy and drug resistance through theregulation of PI3K/mTOR pathway and downregulation of P26 [53].

This study showed a significant association between ARK5 and SIRT3 expression in tumour cells of RCC. The same association has been observed by Xu et al., 2020 in the cancer cervix. SIRT3 was found to regulate AMPK expression in tumour cells and also AMPK was found to have a direct stimulatory effect on SIRT3 expression [54].

The current study revealed a significant association between ARK5 and SIRT3 expression in renal cell carcinoma cases collectively and clear cell type specifically and shorter patients’ survival but none of them was an independent variable. ARK5 expression was associated with poor prognosis in ovarian cancer [55]. Another study showed that knockout of ARK5 was associated with better survival in gastric carcinoma [35]. Zhao et al., 2013 have also documented that SIRT3 expression was associated with worse prognosis and short survival in oesophagal cancer [56].

Briefly, this study is shedding light on the prognostic significance of ARK5 and SIRT3 overexpression in RCCs. Interestingly, nuclear ARK5 expression seems more significant than cytoplasmic expression. Nucleocytoplasmic expression of ARK5 and SIRT3 positivity may have a role in RCC carcinogenesis which is supported by being higher in RCC than non-neoplastic cases. Their expression may promote RCC ability for invasion, metastasis and resistance to therapy. They negatively affect the survival of all RCC types and clear cell type in particular. They may be considered for future targeted therapy in selected cases aiming at modulating tumour behaviour and improving patients' prognosis. However, future testing of the effect of their blocking on the tumor response is recommended. Moreover, a novel postulated link between ARK5 and SIRT3 has been discovered that needs more extensive research on a larger cohort of patients with a larger number of different types of RCC.

The limited number of cases with available paraffin blocks and clinical data was one of the major limitations of the current study. Other limitations include the limited quality of the available microscopes, the lack of digital scoring systems of IHC as well as absence of financial support. Thus, the future research direction is to apply the same research on a larger scale including a variety of RCC types and using digital scoring and functional assays by in-vitro techniques and molecular testing.

## Data Availability

The datasets generated and/or analysed during the current study are available in tables in this article. The raw data is only available for authors and not publicly available due to ethical restrictions.
